# Reliability and validity of the Hospital Anxiety and Depression Scale in an emergency department in Saudi Arabia: a cross-sectional observational study

**DOI:** 10.1186/s12873-015-0051-4

**Published:** 2015-10-12

**Authors:** Zohair A. Al Aseri, M. Owais Suriya, Hosam A. Hassan, Mujtaba Hasan, Shaffi Ahmed Sheikh, Adel Al Tamimi, Mashhoor Alshathri, Najeeb Khalid

**Affiliations:** Department of Emergency Medicine (65), College of Medicine, King Khalid University Hospital KSU, PO Box No: 7805, Riyadh, 11472 Kingdom of Saudi Arabia; Fellow Community Health, College of Medicine, University of Saskatchewan, 107, Wiggins Road, S7N 5E5 Saskatoon, Canada; Department of Family and Community Medicine, College of Medicine King Khalid University Hospital KSU, PO Box 230155, Riyadh, 11321 Kingdom of Saudi Arabia; Cardiff and Vale University Health Board, Cardiff, UK

**Keywords:** Hospital Anxiety and Depression Scale (HADS), Anxiety and depression, Accident and emergency department, Psychiatric comorbidities

## Abstract

**Background:**

Depression and anxiety are prevalent psychiatric comorbidities that are known to have a negative impact on a patient’s general prognosis. But screening for these potential comorbidities in a hospital’s accident and emergency department has seldom been undertaken, particularly in Saudi Arabia and elsewhere in the Middle East. The Hospital Anxiety and Depression Scale (HADS) has been extensively used to evaluate these psychiatric comorbidities in various clinical settings at all levels of health care services except for the accident and emergency department. This study therefore aimed to assess the reliability and validity of the HADS for anxiety and depression among patients at a hospital accident and emergency department in Saudi Arabia.

**Methods:**

This cross-sectional observational study was conducted from January to December 2012. The participants were 257 adult patients (aged 16 years and above) who presented at the accident and emergency department of King Khalid University Hospital, Riyadh, Saudi Arabia, who met our inclusion criteria. We used an Arabic translation of the HADS. We employed factor analysis to determine the underlying factor structure of that instrument in assessing reliability and validity.

**Results:**

We found the Arabic version of the HADS to be acceptable for 95 % of the subjects. We used Cronbach’s alpha coefficient to evaluate reliability, and it indicated a significant correlation with both the anxiety (0.73) and depression (0.77) subscales of the HADS, thereby supporting the validity of the instrument. By means of factor analysis, we obtained a two-factor solution according to the two HADS subscales (anxiety and depression), and we observed a statistically significant correlation (*r* = 0.57; *p* < 0.0001) between the two subscales.

**Conclusion:**

The HADS can be used effectively in an accident and emergency department as an initial screening instrument for anxiety and depression. It thus has great potential as part of integrated multidisciplinary care.

## Background

The prevailing concept of health clearly identifies mind and body as “patho-physiologically inter-dependable entities” that need to be in harmony with the “environment, internal as well as external” to achieve “complete well-being” [1]. The existing literature has identified one aspect of this integral association in terms of the high prevalence of psychiatric comorbidities, especially depressive and anxiety disorders, among medically ill patients [2, 3]. In the Arab world, the prevalence of such psychiatric disorders is quite high, with rates of anxiety and depression being reportedly 26–40 % [4–6]. However, many studies worldwide have consistently found low detection rates of such psychiatric comorbidities by attending physicians, thereby resulting in delays in appropriate diagnoses and treatment [9–11]. These delays probably make a substantial contribution to the burden of illness and to the add-on economic impacts of resulting disabilities—for the patient as well as for the health care system and society in general.

Several questionnaires have been developed as screening tools to detect such psychiatric comorbidities. The Hospital Anxiety and Depression Scale (HADS) was formulated by Zigmond and Sanith [12] to identify possible or probable anxiety and depression among patients in non-psychiatric clinical settings. The HADS anxiety and depression subscales each consist of seven related items. Each item is rated on a four-point scale from 0 to 3, yielding a maximum score of 21 for each subscale. Scores of 11 or more with either subscale are considered to indicate a significant “case” of psychiatric comorbidity; scores of 8–10 signify the presence of a “disorder.” A score of 7 or less is considered normal [13, 14]. Since its inception, this instrument has been used extensively worldwide, and it has been translated into many languages, including German, Swedish, Chinese, French, Dutch, Portuguese [15], Farsi (Persian) [16], and Arabic.

The Arabic version of the HADS has been used in various health care settings, both at primary- and secondary-care levels, in such countries as Saudi Arabia [5, 6], Kuwait [7], and the United Arab Emirates [8]. There, it has been demonstrated to be a valid, reliable screening instrument in a primary-care setting [7, 8]—just as with its versions in other languages [15, 16]. However, it has been rarely used in accident and emergency (A&E) departments in Arab countries; this is in contrast to other parts of the world, where it has been used successfully for screening purposes in such clinical settings [26]. Patients attending an A&E department are generally in a state of distress owing to the acute nature of their health problems, and so it would appear to be highly appropriate to screen these patients for existing psychiatric comorbidities, such as anxiety and depression.

The present study aimed to identify the existence of prevalent psychiatric comorbidities and to evaluate the sensitivity of the Arabic version of the HADS in a hospital A&E department setting in Saudi Arabia. From the perspective of providing holistic care, a further purpose of this study was to increase awareness among health care professionals about the importance of recognizing such psychiatric comorbidities as anxiety and depression in patients attending the A&E department.

## Methods

### Study design, setting, patient recruitment, and data collection

In this cross-sectional observational study, we recruited participants among patients who attended the A&E department of King Khalid University Hospital, Riyadh, Saudi Arabia, from January to December 2012. The Arabic version of the HADS was used after obtaining prior permission from its sole proprietor, GL Assessment of the United Kingdom [7, 8].

The printed HADS questionnaire in Arabic was provided to all patients aged 16 years and above who were assessed to be clinically stable and were able to read and understand the HADS. We excluded from this study patients who were clinically unstable or whose medical records indicated existing psychiatric disorders. The purpose of the study and the HADS questionnaire was fully described to each included patient beforehand. A signed form indicating informed consent for participation was obtained from each patient. Every subject was given 30 min to complete the questionnaire. Patients who signaled difficulty in reading a particular question on the HADS were assisted by the emergency physician on duty. In such instances, the identified questions were read out to the patients without any explanation or elaboration as to the meaning.

We distributed 298 questionnaires and obtained 257 completed responses for subsequent analysis. The remaining 41 responses were excluded either because the patient refused to participate from the outset (29 subjects) or submitted incomplete questionnaires (12 patients). The patients’ demographic data were recorded each time by the A&E department physicians on duty; the data included age, sex, marital status, educational background, economic status, presenting complaints, medical diagnoses, and previous admissions to the hospital or A&E department visits.

### Ethical approval

The study was formally approved by the Institutional Review Board of the College of Medicine, King Khalid University Hospital.

### Statistical analysis

We analyzed the data using SPSS software, version 16.0 for Windows (released 2007; Chicago, IL, USA). We assessed internal consistency using Cronbach’s alpha; convergent validity was evaluated using Pearson’s correlation coefficient among the items, subscale scores, and total scores. We further determined the validity of the HADS by means of factor analysis, in which the correlation matrix, Kaiser–Meyer–Olkin measurement of sampling adequacy, and Bartlett’s test of sphericity were used to assess the factorability of the 14 questionnaire items. We examined the factor structure of the HADS by restricting the factor extraction process to two factors and employing the principal component method. Proportion of variance was assessed through initial eigenvalues explained by each of the factors. We used varimax rotation to obtain the rotated factors. Pearson’s chi-square test was utilized to assess the associations between the categorical study variables and the three levels of depression and anxiety. We employed a *p* value of <0.05 to determine the statistical significance of the study results.

## Results

Of the 257 patients, 105 (40.9 %) were aged 16–30 years; 115 (44.7 %) were male. A high literacy status was held by 25 patients (9.7 %); 148 (57.6 %) had attended only primary or secondary school; the remaining 84 (32.7 %) patients had no formal education. With respect to marital status, 139 (54.1 %) patients were married, 84 (32.7 %) had never married, and the remaining 34 (13.2 %) were divorced or widowed.

Factor analysis of the correlation matrix showed that 12 of the 14 questionnaire items correlated (at least 0.30) with other items, which suggests reasonable factorability. Further, the Kaiser–Meyer–Olkin measurement of sampling adequacy was 0.864—greater than the recommended value of 0.6; Bartlett’s test of sphericity was also significant: *χ*^2^_(91)_ =917.57, *p* < 0.0001. In addition, the communalities were all greater than 0.45, which indicates that each item was included in the factor analysis. The initial eigenvalues showed that first factor extracted by the principal component method explained about 22.8 % of the total variance; the second factor accounted for 21.36 % of the variance. The rotated factor structure indicated a two-factor structure that was closely related to the anxiety and depression subscales. Seven items were loaded onto the first component of the factor structure, with a factor loading range of 0.813–0.284. That factor was loaded with all six items of the anxiety subscale, but the seventh item (“I can sit at ease and feel relaxed,” which had a factor loading of 0.629) was loaded with the second factor on the depression subscale. The remaining seven items were loaded onto the second component of the factor structure, with a factor loading range of 0.717–0.527. Of those seven items, six were loaded with the depression subscale; the remaining item (“I feel as though I have slowed down,” which had a factor loading of 0.52) was loaded with the first factor on the anxiety subscale (Table 1).

**Table 1:** Factor analysis of the HADS items using varimax rotation

Item No.	Subscale	Item	1	2
13	Anxiety	I get a sudden feeling of panic.	0.813	0.103
9	Anxiety	I get a sort of frightened feeling like ‘butterflies’ in the stomach.	0.736	0.051
3	Anxiety	I get a sort of frightened feeling as if something awful is about to happen.	0.721	0.109
5	Anxiety	Worrying thoughts go through my mind.	0.684	0.167
1	Anxiety	I feel tense or wound up.	0.518	0.335
11	Anxiety	I feel restless as if I have to be on the move.	0.284	0.259
7	Depression	I can sit and feel relaxed.	0.085	0.629
12	Depression	I look forward with enjoyment to things.	0.141	0.717
2	Depression	I still enjoy the things I used to enjoy.	0.144	0.663
4	Depression	I can laugh and see the funny sides of things.	0.141	0.656
10	Depression	I have lost interest in my appearance.	0.131	0.626
14	Depression	I can enjoy a good book or radio or TV program.	0.141	0.583
6	Depression	I feel cheerful.	0.261	0.560
8	Anxiety	I feel disappointed (or feel slowed down).	0.527	0.368

Rotation converged in three iterations

Cronbach’s alpha for the instrument was 0.83, with alpha values of 0.73 and 0.77 for the anxiety and depression subscales, respectively. Analyses of the correlation of each item with its hypothesized scale revealed the Pearson’s correlation coefficients to be 0.49–0.73 for the anxiety subscale and 0.56–0.71 for the depression subscale. All those correlations were statistically significant (*p* < 0.0001). In addition, significant intercorrelations were observed between the following: the anxiety and depression subscales (*r* = 0.57; *p* < 0.001); the anxiety subscale and the HADS (*r* = 0.88; *p* < 0.001); and the depression subscale and the HADS (*r* = 0.90; *p* < 0.001; Table 2).

**Table 2:** Correlation of the HADS items with their hypothesized subscales and HADS overall

	HADS-A (anxiety subscale)	HADS-D (depression subscale)	HADS (overall)
*Item number/Anxiety (HADS-A)*	*1*	*0.572*	*0.88*
13/I get sudden feeling of panic.	0.734	0.358	0.529
9/I get a sort of frightened feeling like ‘butterflies’ in the stomach.	0.660	0.284	0.581
3/I get a sort of frightened feeling as if something awful is about to happen.	0.673	0.345	0.582
5/Worrying thoughts go through my mind.	0.674	0.370	0.566
1/I feel tense or wound up.	0.640	0.408	0.584
11/I feel restless as if I have to be on the move.	0.504	0.257	0.584
8/I feel as if I am slowed down.	0.533	0.640	0.420
*Item number/Depression (HADS-D)*	*0.572*	*1*	*0.90*
7/I can sit at ease and feel relaxed.	0.486	0.459	0.577
12/I look forward with enjoyment to things.	0.386	0.666	0.607
2/I still enjoy the things I used to enjoy.	0.359	0.667	0.616
4/I can laugh and see the funny side of things.	0.352	0.664	0.524
10/I have lost interest in my appearance.	0.308	0.562	0.622
14/I can enjoy a good book or TV program.	0.305	0.711	0.550
6/I feel cheerful.	0.416	0.620	0.554

Pearson’s correlation coefficient and all differences were significant at the 0.01 level (*p* < 0.0001)

Our study used the cut-off values recommended by the author of the HADS: scores greater than 11 on either subscale were considered significant cases of psychiatric morbidity; scores of 8–10 signified the presence of disorders; scores of 7 or less were considered normal. Using these cut-off values for the total scores for the two subscales of the HADS, we found the rates of depression and anxiety to be 27.2 and 23 %, respectively.

## Discussion

It is feasible to use a cross-sectional approach to interpret data relating to the translation of a screening tool [5–8, 15, 16]. The HADS is an extensively used psychometric tool that has been utilized directly and in translated versions in various languages. The Arabic version of the HADS has been tested in a number of clinical and community settings [5–8]; however, it has hitherto seldom been employed in an A&E department setting. The present study is the first to apply the HADS in an Arabic context: the translation was well accepted by the patients, which is in accordance with the previously reported high levels of general acceptability of the HADS [7, 8, 15]. In contrast with the results for some other translated versions [16, 17], we identified no problems with any of the HADS items in the Arabic version [7, 8].

We found the results of the internal consistency and confirmatory factor analyses to be significant: they appear to underline the reliability and validity of the Arabic version of the HADS in the context of an A&E department. As with the results of other studies [16, 18], the present investigation observed a strong correlation between the anxiety and depression subscales. This correlation may have been the result of similarities between anxiety and depression in terms of symptomatology as well as in their postulated background biopsychosocial etiological models. The validity analyses of the Arabic version of the HADS revealed a two-factor structure, in which six items in each of the two factors were loaded correctly, but one item for each factor was loaded in the opposite manner. This structure may be due to intercorrelation between the anxiety and depression subscales; it is very unlikely to be the result of flaws in the items in the HADS instrument. These findings substantiate the basis for the two-factor structure for the Arabic version of the HADS, and they are in line with the results of other studies [17, 19–24].

In the present study, we found the overall occurrence of anxiety to be 27.2 %, that of depression 23.0 %. The rates of these psychiatric comorbidities in a hospital A&E department are different from those reported in other studies and may be due to the use of different cut-off scores. Another potential contributing factor may be the present study having adopted a wider nonspecific patient pool, i.e., all patients presenting to the A&E department with any complaints. By contrast, most other similar studies investigating A&E departments focused primarily on patients presenting with one particular complaint. For example, a study conducted in Brazil examined patients with chest pain: it reported a higher occurrence rate for anxiety of 33.9 % and for depression of 30.5 % [25]. In an Indian study, the rate of combined anxiety and depression was determined to be 23 % among patients who presented to the A&E department of a university hospital with chest pain. That is very similar to the results found in the present study [26]. However, one Turkish study of an A&E department observed anxiety in 38.1 % of patients with non-cardiac chest pain compared with 40 % of patients with cardiac chest pain; depression was observed in 52.3 % of cardiac chest pain patients versus 52.1 % of non-cardiac chest pain patients [27]. Those findings are higher than the results obtained in the present study. However, a similar Turkish study observed anxiety in 23 % of patients with chest pain, which is lower than the frequency observed in this investigation [28]. A very high occurrence of anxiety (up to 73.3 %) was noted in another study, which focused on patients who presented at an A&E department with nonspecific chest pain; [29] that is in contrast to the lower reported frequency of combined anxiety and depression among 21 % of patients who presented at an A&E department following substance abuse. A recent study in France assessing the proficiency of physicians in detecting anxiety and depression among the general pool of patients who presented at an A&E department found the occurrence of anxiety to be 47 % and that of depression to be 27 % [19].

This study has a few limitations that deserve mention. One of these was including only subjects who were found to be clinically stable; thus, the results cannot be regarded as being truly representative of all patients presenting at a hospital A&E department. In addition, owing to the clinical context in the present study, it was not feasible to conduct a follow-up evaluation of positively screened cases by a psychiatrist toward further testing the sensitivity and specificity of the HADS. However, our findings do provide evidence regarding the underlying two-factor structure of the HADS.

## Conclusions

To the best of our knowledge, the present study is the first to be based on factor analysis in the subspecialty of an A&E department using the Arabic translation of the HADS. This study found that despite the hectic working environment of an A&E department, the HADS performed well in screening for the two most prevalent psychiatric comorbidities—anxiety and depression. Our findings underscore the merits of the HADS—especially in light of the well-established fact that non-psychiatrist physicians have less ability to screen for psychiatric conditions in general. However, efficient processes and protocol still need to be developed in the Arab context for prompt follow-up psychiatric assessments of cases identified using the HADS so to ensure integrated care planning from a more holistic perspective.
