# Correction to: Reliability and validity of the Hospital Anxiety and Depression Scale in an emergency department in Saudi Arabia: a cross-sectional observational study

**DOI:** 10.1186/s12873-020-0305-7

**Published:** 2020-01-31

**Authors:** Zohair A. Al Aseri, M. Owais Suriya, Hosam A. Hassan, Mujtaba Hasan, Shaffi Ahmed Sheikh, Adel Al Tamimi, Mashhoor Alshathri, Najeeb Khalid

**Affiliations:** 1 Departments of Emergency Medicine and Critical Care, King Saud University, College of Medicine, Riyadh, Saudi Arabia; 2 Fellow Community Health, College of Medicine, University of Saskatchewan, 107, Wiggins Road, Saskatoon, S7N 5E5 Canada; 3 Department of Family and Community Medicine, College of Medicine King Khalid University Hospital KSU, PO Box 230155, Riyadh, 11321 Kingdom of Saudi Arabia; 4 Cardiff and Vale University Health Board, Cardiff, UK

**Correction to: BMC Emerg Med**

**https://doi.org/10.1186/s12873-015-0051-4**

The original article [1] contains an incorrect affiliation. Affiliation #1 should instead read as the following:

- *Departments of Emergency Medicine and Critical Care, King Saud University, College of Medicine, Riyadh, Saudi Arabia.*
